# Supplementary material for: A predictive model for canine dilated cardiomyopathy—a meta-analysis of Doberman Pinscher data
Source: PeerJ. 2015 Mar 26;3:e842. doi: 10.7717/peerj.842 (PMC4380154; doi:10.7717/peerj.842)
Supplement: Table S1 [file peerj-03-842-s002.docx]

# Supplementary Table 1. The phenotype decisions, combined genotype frequencies and predicted number of individuals for each genotype combination from the model incorporating the two known DCM loci.

| **PDK4** | | **Chr5 SNP** | | **Combined genotype freq** | **Predicted number of individuals** | **Phenotype** |
| --- | --- | --- | --- | --- | --- | --- |
| **genotype** | **freq** | **genotype** | **freq** |  |  |  |
| Wt Wt | 0.72 | TT | 0.74 | 0.5328 | 96.9696 | Healthy |
| Wt Wt | 0.72 | TC | 0.24 | 0.1728 | 31.4496 | Healthy |
| Wt Wt | 0.72 | CC | 0.02 | 0.0144 | 2.6208 | DCM |
| Wt del | 0.26 | TT | 0.74 | 0.1924 | 35.0168 | Healthy |
| Wt del | 0.26 | TC | 0.24 | 0.0624 | 11.3568 | DCM |
| Wt del | 0.26 | CC | 0.02 | 0.0052 | 0.9464 | DCM |
| Del del | 0.02 | TT | 0.74 | 0.0148 | 2.6936 | Healthy |
| Del del | 0.02 | TC | 0.24 | 0.0048 | 0.8736 | DCM |
| Del del | 0.02 | CC | 0.02 | 0.0004 | 0.0728 | DCM |
