# Supplementary material for: A predictive model for canine dilated cardiomyopathy—a meta-analysis of Doberman Pinscher data
Source: PeerJ. 2015 Mar 26;3:e842. doi: 10.7717/peerj.842 (PMC4380154; doi:10.7717/peerj.842)
Supplement: Table S2 [file peerj-03-842-s003.docx]

**Supplementary Table 2.** The phenotype decisions, combined genotype frequencies and predicted number of individuals for each genotype combination incorporating the two known DCM loci + 50% (males) more susceptible to DCM.

| **sex** | | **PDK4** | | **Chr5 SNP** | | **combined genotype freq** | **Predicted number of individuals** | **Phenotype** |
| --- | --- | --- | --- | --- | --- | --- | --- | --- |
| **sex** | **freq** | **genotype** | **freq** | **genotype** | **freq** |  |  |  |
| female | 0.5 | Wt Wt | 0.72 | TT | 0.74 | 0.2664 | 48.4848 | Healthy |
| female | 0.5 | Wt Wt | 0.72 | TC | 0.24 | 0.0864 | 15.7248 | Healthy |
| female | 0.5 | Wt Wt | 0.72 | CC | 0.02 | 0.0072 | 1.3104 | DCM |
| female | 0.5 | Wt del | 0.26 | TT | 0.74 | 0.0962 | 17.5084 | Healthy |
| female | 0.5 | Wt del | 0.26 | TC | 0.24 | 0.0312 | 5.6784 | DCM |
| female | 0.5 | Wt del | 0.26 | CC | 0.02 | 0.0026 | 0.4732 | DCM |
| female | 0.5 | Del del | 0.02 | TT | 0.74 | 0.0074 | 1.3468 | Healthy |
| female | 0.5 | Del del | 0.02 | TC | 0.24 | 0.0024 | 0.4368 | DCM |
| female | 0.5 | Del del | 0.02 | CC | 0.02 | 0.0002 | 0.0364 | DCM |
| male | 0.5 | Wt Wt | 0.72 | TT | 0.74 | 0.2664 | 48.4848 | Healthy |
| male | 0.5 | Wt Wt | 0.72 | TC | 0.24 | 0.0864 | 15.7248 | DCM |
| male | 0.5 | Wt Wt | 0.72 | CC | 0.02 | 0.0072 | 1.3104 | DCM |
| male | 0.5 | Wt del | 0.26 | TT | 0.74 | 0.0962 | 17.5084 | DCM |
| male | 0.5 | Wt del | 0.26 | TC | 0.24 | 0.0312 | 5.6784 | DCM |
| male | 0.5 | Wt del | 0.26 | CC | 0.02 | 0.0026 | 0.4732 | DCM |
| male | 0.5 | Del del | 0.02 | TT | 0.74 | 0.0074 | 1.3468 | DCM |
| male | 0.5 | Del del | 0.02 | TC | 0.24 | 0.0024 | 0.4368 | DCM |
| male | 0.5 | Del del | 0.02 | CC | 0.02 | 0.0002 | 0.0364 | DCM |
