# Supplementary material for: A predictive model for canine dilated cardiomyopathy—a meta-analysis of Doberman Pinscher data
Source: PeerJ. 2015 Mar 26;3:e842. doi: 10.7717/peerj.842 (PMC4380154; doi:10.7717/peerj.842)
Supplement: Table S4 [file peerj-03-842-s005.docx]

**Supplementary Table 4.** Phenotype decisions, genotype frequencies and predicted number of individuals for each genotype combination incorporating the two known DCM loci + an autosomal additive DCM susceptibility locus.

| **additional autosomal additive locus** | | **PDK4** | | **Chr5 SNP** | | **combined genotype freq** | **Predicted number of individuals** | **Phenotype** |
| --- | --- | --- | --- | --- | --- | --- | --- | --- |
| **genotype** | **freq** | **genotype** | **freq** | **genotype** | **freq** |  |  |  |
| good good | 0.490 | Wt Wt | 0.72 | TT | 0.74 | 0.261072 | 47.515104 | Healthy |
| good good | 0.490 | Wt Wt | 0.72 | TC | 0.24 | 0.084672 | 15.410304 | Healthy |
| good good | 0.490 | Wt Wt | 0.72 | CC | 0.02 | 0.007056 | 1.284192 | DCM |
| good good | 0.490 | Wt del | 0.26 | TT | 0.74 | 0.094276 | 17.158232 | Healthy |
| good good | 0.490 | Wt del | 0.26 | TC | 0.24 | 0.030576 | 5.564832 | DCM |
| good good | 0.490 | Wt del | 0.26 | CC | 0.02 | 0.002548 | 0.463736 | DCM |
| good good | 0.490 | Del del | 0.02 | TT | 0.74 | 0.007252 | 1.319864 | Healthy |
| good good | 0.490 | Del del | 0.02 | TC | 0.24 | 0.002352 | 0.428064 | DCM |
| good good | 0.490 | Del del | 0.02 | CC | 0.02 | 0.000196 | 0.035672 | DCM |
| good bad | 0.420 | Wt Wt | 0.72 | TT | 0.74 | 0.223776 | 40.727232 | Healthy |
| good bad | 0.420 | Wt Wt | 0.72 | TC | 0.24 | 0.072576 | 13.208832 | DCM |
| good bad | 0.420 | Wt Wt | 0.72 | CC | 0.02 | 0.006048 | 1.100736 | DCM |
| good bad | 0.420 | Wt del | 0.26 | TT | 0.74 | 0.080808 | 14.707056 | DCM |
| good bad | 0.420 | Wt del | 0.26 | TC | 0.24 | 0.026208 | 4.769856 | DCM |
| good bad | 0.420 | Wt del | 0.26 | CC | 0.02 | 0.002184 | 0.397488 | DCM |
| good bad | 0.420 | Del del | 0.02 | TT | 0.74 | 0.006216 | 1.131312 | DCM |
| good bad | 0.420 | Del del | 0.02 | TC | 0.24 | 0.002016 | 0.366912 | DCM |
| good bad | 0.420 | Del del | 0.02 | CC | 0.02 | 0.000168 | 0.030576 | DCM |
| bad bad | 0.090 | Wt Wt | 0.72 | TT | 0.74 | 0.047952 | 8.727264 | DCM |
| bad bad | 0.090 | Wt Wt | 0.72 | TC | 0.24 | 0.015552 | 2.830464 | DCM |
| bad bad | 0.090 | Wt Wt | 0.72 | CC | 0.02 | 0.001296 | 0.235872 | DCM |
| bad bad | 0.090 | Wt del | 0.26 | TT | 0.74 | 0.017316 | 3.151512 | DCM |
| bad bad | 0.090 | Wt del | 0.26 | TC | 0.24 | 0.005616 | 1.022112 | DCM |
| bad bad | 0.090 | Wt del | 0.26 | CC | 0.02 | 0.000468 | 0.085176 | DCM |
| bad bad | 0.090 | Del del | 0.02 | TT | 0.74 | 0.001332 | 0.242424 | DCM |
| bad bad | 0.090 | Del del | 0.02 | TC | 0.24 | 0.000432 | 0.078624 | DCM |
| bad bad | 0.090 | Del del | 0.02 | CC | 0.02 | 0.000036 | 0.006552 | DCM |
