# Supplementary material for: A predictive model for canine dilated cardiomyopathy—a meta-analysis of Doberman Pinscher data
Source: PeerJ. 2015 Mar 26;3:e842. doi: 10.7717/peerj.842 (PMC4380154; doi:10.7717/peerj.842)
Supplement: Table S6 — Results from the model incorporating the two known DCM loci + an additional X-linked DCM susceptibility locus, where X is normal and x is susceptible. [file peerj-03-842-s007.docx]

**Supplementary Table 6.** The phenotype decisions, combined genotype frequencies and predicted number of individuals for each genotype combinations.

| **additional X locus** | | **PDK4** | | **Chr5 SNP** | | **combined genotype freq** | **Predicted number of individuals** | **Phenotype** |
| --- | --- | --- | --- | --- | --- | --- | --- | --- |
| **genotype** | **freq** | **genotype** | **freq** | **genotype** | **freq** |  |  |  |
| XY | 0.350 | Wt Wt | 0.72 | TT | 0.74 | 0.18648 | 33.93936 | Healthy |
| XY | 0.350 | Wt Wt | 0.72 | TC | 0.24 | 0.06048 | 11.00736 | Healthy |
| XY | 0.350 | Wt Wt | 0.72 | CC | 0.02 | 0.00504 | 0.91728 | DCM |
| XY | 0.350 | Wt del | 0.26 | TT | 0.74 | 0.06734 | 12.25588 | Healthy |
| XY | 0.350 | Wt del | 0.26 | TC | 0.24 | 0.02184 | 3.97488 | DCM |
| XY | 0.350 | Wt del | 0.26 | CC | 0.02 | 0.00182 | 0.33124 | DCM |
| XY | 0.350 | Del del | 0.02 | TT | 0.74 | 0.00518 | 0.94276 | Healthy |
| XY | 0.350 | Del del | 0.02 | TC | 0.24 | 0.00168 | 0.30576 | DCM |
| XY | 0.350 | Del del | 0.02 | CC | 0.02 | 0.00014 | 0.02548 | DCM |
| xY | 0.150 | Wt Wt | 0.72 | TT | 0.74 | 0.07992 | 14.54544 | DCM |
| xY | 0.150 | Wt Wt | 0.72 | TC | 0.24 | 0.02592 | 4.71744 | DCM |
| xY | 0.150 | Wt Wt | 0.72 | CC | 0.02 | 0.00216 | 0.39312 | DCM |
| xY | 0.150 | Wt del | 0.26 | TT | 0.74 | 0.02886 | 5.25252 | DCM |
| xY | 0.150 | Wt del | 0.26 | TC | 0.24 | 0.00936 | 1.70352 | DCM |
| xY | 0.150 | Wt del | 0.26 | CC | 0.02 | 0.00078 | 0.14196 | DCM |
| xY | 0.150 | Del del | 0.02 | TT | 0.74 | 0.00222 | 0.40404 | DCM |
| xY | 0.150 | Del del | 0.02 | TC | 0.24 | 0.00072 | 0.13104 | DCM |
| xY | 0.150 | Del del | 0.02 | CC | 0.02 | 0.00006 | 0.01092 | DCM |
| **Females (XX)** |  | **PDK4** |  | **Chr5 SNP** |  |  |  | **Phenotype** |
| XX | 0.25 | Wt Wt | 0.72 | TT | 0.74 | 0.1332 | 24.2424 | Healthy |
| XX | 0.25 | Wt Wt | 0.72 | TC | 0.24 | 0.0432 | 7.8624 | Healthy |
| XX | 0.25 | Wt Wt | 0.72 | CC | 0.02 | 0.0036 | 0.6552 | DCM |
| XX | 0.25 | Wt del | 0.26 | TT | 0.74 | 0.0481 | 8.7542 | Healthy |
| XX | 0.25 | Wt del | 0.26 | TC | 0.24 | 0.0156 | 2.8392 | DCM |
| XX | 0.25 | Wt del | 0.26 | CC | 0.02 | 0.0013 | 0.2366 | DCM |
| XX | 0.25 | Del del | 0.02 | TT | 0.74 | 0.0037 | 0.6734 | Healthy |
| XX | 0.25 | Del del | 0.02 | TC | 0.24 | 0.0012 | 0.2184 | DCM |
| XX | 0.25 | Del del | 0.02 | CC | 0.02 | 0.0001 | 0.0182 | DCM |
| Xx | 0.210 | Wt Wt | 0.72 | TT | 0.74 | 0.111888 | 20.363616 | Healthy |
| Xx | 0.210 | Wt Wt | 0.72 | TC | 0.24 | 0.036288 | 6.604416 | DCM |
| Xx | 0.210 | Wt Wt | 0.72 | CC | 0.02 | 0.003024 | 0.550368 | DCM |
| Xx | 0.210 | Wt del | 0.26 | TT | 0.74 | 0.040404 | 7.353528 | DCM |
| Xx | 0.210 | Wt del | 0.26 | TC | 0.24 | 0.013104 | 2.384928 | DCM |
| Xx | 0.210 | Wt del | 0.26 | CC | 0.02 | 0.001092 | 0.198744 | DCM |
| Xx | 0.210 | Del del | 0.02 | TT | 0.74 | 0.003108 | 0.565656 | DCM |
| Xx | 0.210 | Del del | 0.02 | TC | 0.24 | 0.001008 | 0.183456 | DCM |
| Xx | 0.210 | Del del | 0.02 | CC | 0.02 | 0.000084 | 0.015288 | DCM |
| xx | 0.050 | Wt Wt | 0.72 | TT | 0.74 | 0.02664 | 4.84848 | DCM |
| xx | 0.050 | Wt Wt | 0.72 | TC | 0.24 | 0.00864 | 1.57248 | DCM |
| xx | 0.050 | Wt Wt | 0.72 | CC | 0.02 | 0.00072 | 0.13104 | DCM |
| xx | 0.050 | Wt del | 0.26 | TT | 0.74 | 0.00962 | 1.75084 | DCM |
| xx | 0.050 | Wt del | 0.26 | TC | 0.24 | 0.00312 | 0.56784 | DCM |
| xx | 0.050 | Wt del | 0.26 | CC | 0.02 | 0.00026 | 0.04732 | DCM |
| xx | 0.050 | Del del | 0.02 | TT | 0.74 | 0.00074 | 0.13468 | DCM |
| xx | 0.050 | Del del | 0.02 | TC | 0.24 | 0.00024 | 0.04368 | DCM |
| xx | 0.050 | Del del | 0.02 | CC | 0.02 | 0.00002 | 0.00364 | DCM |

Results from the model incorporating the two known DCM loci + an additional X-linked DCM susceptibility locus, where X is normal and x is susceptible.
